# Final diagnoses and probability of new reason-for-encounter at an urban clinic in Japan: A 4-year observational study

**DOI:** 10.1097/MD.0000000000006999

**Published:** 2017-06-02

**Authors:** Motoharu Fukushi, Yukishige Ishibashi, Naoki Nago

**Affiliations:** a Musashi Kokubunji Park Clinic (Jikkoukai Medical Corporation); b Ishibashi Clinic (Jikkoukai Medical Corporation), Tokyo, Japan.

**Keywords:** Bayes theorem, diagnosis, medical coding, observational study, reason-for-encounter

## Abstract

Past clinical data are not currently used to calculate pretest probabilities, as they have not been put into a database in clinical settings. This observational study was designed to determine the initial reasons for utilizing home visits or visits to an outpatient urban clinic in Japan.

All family medical clinic outpatients and patients visited by the clinic (total = 11,688) over 1460 days were enrolled.

We used a Bayes theorem-based clinical decision support system to analyze codes for initial reason-for-encounter (examination and final diagnosis: pretest probability) and final diagnosis of patients with fever (conditional pretest probability).

Total number of reasons-for-encounter: 96,653 (an average of 1.2 reasons per visit). Final diagnosis: 62,273 cases (an average of 0.75 cases per visit). The most common reasons for initial examination were immunizations, physical examinations, and upper respiratory conditions. Regarding the final diagnosis, the combination of physical examinations and acute upper respiratory infections comprised 73.4% of cases. In cases where fever developed, the bulk of the final diagnoses were infectious diseases such as influenza, strep throat, and gastroenteritis of presumed infectious origin. For the elderly, fever often occurred with other health issues such as pneumonia, dementia, constipation, and sleep disturbances, though the cause of the fever remained undetermined in 40% of the cases.

The pretest probability changed significantly based on the reason or the combination of reasons for which patients requested a medical examination. Using accumulated data from past diagnoses to modify subsequent subjective diagnoses, individual diagnoses can be improved.

## 1 Introduction

What is happening in the examination room? Though there is a vague understanding based on experience of the overall picture of what takes place, in terms of quantified data, there is an insufficient grasp of what really takes place.

In the 1990s, it was suggested that statistical data could be used in diagnosis if the probability theory proposed by Thomas Bayes in the 1700s (hereafter referred as Bayes theorem) was applied.^[1,2]^ According to Bayes theorem, to estimate a patient's chances of being diagnosed with any disease, it is necessary to obtain the patient's pretest probability and likelihood ratio.^[2,3]^ The likelihood ratio can be rationally calculated using the given sensitivity and specificity, which can be obtained from general observations concerning diagnosis. However, it is necessary to use the statistical data collected from all past diagnoses in that particular medical facility to assess the frequency of a given disease being diagnosed to determine the pretest probability; it would be difficult to obtain such information from a general database of electronic medical records.

Furthermore, to calculate the pretest probability of a patient who had come into the clinic, it is necessary to extract the data regarding the final diagnosis under relevant conditions. However, though past observational studies^[4–12]^ have considered this based on the size of the medical facilities, regional characteristics, and physician skills, they have not sought to determine the relationship between the patient's chief complaint and the final diagnosis or its pretest probability.

Not only does the pretest probability change based on the examination location, such as the size of the medical facility or its specific location, a variety of conditions can affect the outcome of the probability; such conditions include the patient's age, whether or not there is a current epidemic of a given infection, or the time of the day when the patient is being examined. To be able to utilize ever-changing, dynamic statistical data in a clinical setting while providing medical care, there must be a feature within the electronic medical records that collects and analyzes all past diagnostic data and allows real-time referencing. Though there is one such reported case of an electronic medical record that has such a feature, it was developed for research purposes,^[13]^ and no reports of implementation of such a system in actual clinical settings could be found.

To this day, clinicians diagnose their patients with subjective and arbitrary pretest probabilities; this is a concern as it means the individual experience of the clinicians could severely affect their estimates. Using such subjective pretest probabilities is fraught with danger, as it may lead to deciding on the wrong treatment or may worsen the prognosis.

Therefore, we have implemented a clinical decision support system (CDSS), which has as its basic function the ability to reference pretest probabilities based on all past medical data. The CDSS could be used to determine the pretest probability of clinical diagnoses based on all medical data of a family medical clinic.

The main purpose of this study was to diagnose all subjects who used a certain urban clinic using CDSS and determine (1) the initial reasons for patients seeking care (new encounters) and their final diagnosis (pretest probability) based on their age group and (2) the final diagnosis (conditional pretest probability) of patients with fever.

## 2 Methods

### 2.1 Study design

This was an observational study including a descriptive epidemiological study of the frequency of the reason-for-encounter and the final diagnosis (pretest probability) and a cross-sectional study regarding such diagnoses. This study was approved by the institutional review board.

### 2.2 Setting

The study took place in a newly established bed-less family medical clinic on the west side of Tokyo (hereafter referred to as the clinic). Because home visits were conducted, the setting included not only the clinic's examination room but also the residences of the patients as well as facilities for the elderly. The data collection period was 1460 days from the day the clinic opened its doors (June 1, 2011) to the date of analysis (May 31, 2015). There were 14 doctors (hereafter referred to as attending physicians) who attended to the patients during the study. Physicians in their initial clinical training and short-term medical interns were not included.

### 2.3 Participants

All outpatients to the clinic and the patients visited by the clinic for all reasons during the data collection period were the subjects. Therefore, this study includes visits that were only for physical examinations or immunizations.

The participants who came to the clinic for the first time to be examined were told that the patient's reason-for-encounter and diagnostic codes were collected anonymously and utilized for diagnoses, and the overview was explained in the inquiry system and then orally if there was any need to do so. Additionally, we posted a notice in the waiting room explaining that we would use the information for research purposes.

### 2.4 Data source

Age, sex, ID, day of visit, new reason-for-encounter codes, and diagnostic codes collected during the data collection period were entered into CDSS “Dr. Bayes” (Ver. 1, Windows Edition, Macros Japan Co., Ltd.), which was created by the present authors. These data were subsequently used for the analysis. The data were stored in a server computer in the clinic, and all of the analyses were conducted by delinking the patient's name in a way that the data could not be linked back to the specific patient.

The reason-for-encounter code and diagnostic codes conformed to the Japanese version of the International Classification of Primary Care, 2nd Edition (ICPC-2).^[14]^ The reason-for-encounter codes were entered by the participants themselves or by those who accompanied them, such as a family member, directly into a Dr. Bayes through a touch panel by selecting (up to 2) answers in its inquiry system. When the information was gathered by the nurses or the staff or when the participants or their companions reported a condition while receiving care, the attending physicians entered the information at their own discretion. Data regarding all apparent current health issues, such as diseases for which they were being treated, were entered.

The diagnostic codes were entered by the attending physicians based on clinical decisions, at the end of each visit. (No diagnostic criteria were set, and it did not matter whether or not the diagnosis was confirmed through tests.) If a diagnosis was not made by the end of the visit based on the ICPC-2 protocol, the reason-for-encounter code was used as the diagnostic code.

If there were any outcomes, such as recovery, change of attending physician, hospitalization, or death, the attending physician entered the outcome information at the point the information became available. Until all health issues were resolved and the outcome was entered, every visit was considered and treated as 1 episode group. Even within the same episode group, if there were any new reasons-for-encounter or if a diagnosis was changed, the codes were added or changed in each case (Table 1).

**Table 1:**
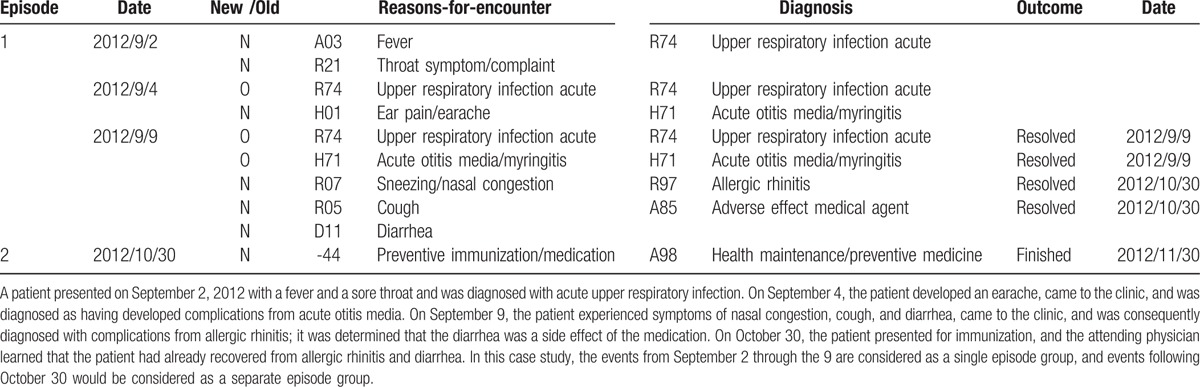
Example of specific codes (fictitious case).

The final diagnosis within each episode group was defined as the final diagnostic code that was entered at the point of the outcome; that is, upon resolution of the health problem, end of treatment, or death, the most recent diagnostic codes on the day of the analysis which were entered at the last visit (or if seen multiple times on the same day, codes were entered when each visit finished).

The diagnostic codes were displayed in the Dr. Bayes unresolved/acute/active column and utilized as a problem list. The attending physicians could decide to move chronic diagnostic codes to the chronic/inactive column at their discretion. However, unless there was an outcome such as recovery, discontinuation, or death, they were tallied as ongoing health issues. Ongoing, chronic, or inactive issues were excluded from the current analysis.

To prevent the attending physicians from using mismatching codes, we added search features in Dr. Bayes to look up ICPC-2 codes as well as a way to display, in order of frequency, the entry codes from past diagnostic data based on sex or age group. If, for example, the attending physicians found that the codes did not align, or if they were unable to determine the relevant codes, they sought each other's opinions and shared information.

### 2.5 Statistical methods

All information within the Dr. Bayes database was extracted based on the following 3 age groups: children (under 15 years), adults (over 15, under 65 years), and elderly (over 65 years). New reasons-for-encounter were displayed based on the number of cases and the ratio (number of cases/all new reasons-for-encounter code numbers) based on the cumulative percentage in order of frequency. The final diagnoses were displayed by the number of cases and pretest probability (number of cases/number of episode groups) in order of frequency.

When the reason-for-encounter displayed A03 Fever in the code, the final diagnoses of such patients were also displayed per age group based on the number of cases, conditional pretest probability (number of cases/number of episode groups that included A03) in order of frequency. Additionally, in the case of children, we displayed not only the A03 Fever code but also any of the cases that expressed R05 Cough, R21 Throat symptom/complaint, S06 Rash localized, D06 Abdominal pain localized other, D10 Vomiting, and D11 Diarrhea in the symptoms; these were displayed based on the number of cases and conditional pretest probability (number of cases/the number of episode groups that included the relevant codes) per age group in order of frequency. The changes in the conditional pretest probability were also expressed with a positive likelihood ratio (LR+) as well.

For the current study, we defined the pretest probability by calculating the number of episode groups as the denominator. This was to more properly reflect the corrections being made on the diagnoses for still open cases. Alternatively, because of the possibility of multiple entries pertaining to the same reasons-for-encounter within 1 episode group, there was a tendency for the pretest probability to be calculated as slightly higher than the actual value.

## 3 Results

### 3.1 Participants

A total of 11,688 participants used the services of the clinic until the day of the analysis. The total number of times our services were used was 83,523 (average use, 7.1 times per person); in 46.8% of cases, the services were used by those under 15 years (Table 2). There were 36,706 episode groups (average, 3.1 episode groups per person).

**Table 2:**
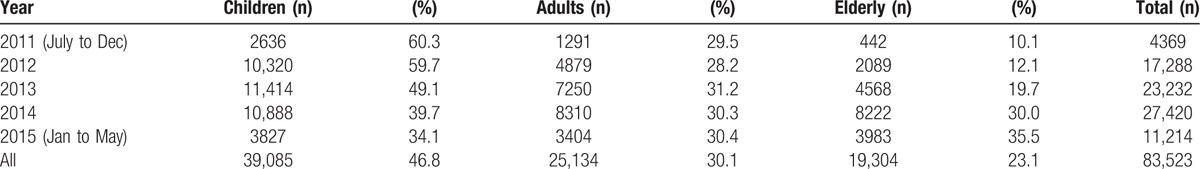
Number of participants.

There were 96,653 codes of new reasons-for-encounter (average, 1.2 cases per visit and 2.63 cases per episode). There were 62,273 codes of final diagnosis (average, 0.75 cases per visit and 1.70 cases per episode). For each case of final diagnosis, there was an average of 1.4 cases of reasons-for-encounter.

### 3.2 Main results: (1) descriptive data and pretest probability

New reasons-for-encounter and final diagnoses are displayed by age group in Tables 3 and 4, respectively. In the new reasons-for-encounter, in all age groups, immunization, physical examination, and upper respiratory conditions were the dominant reasons. In the final diagnosis, A98 Health maintenance/preventive medicine accounted for 26.2% of all diagnoses, and at 44.4%, it had the highest pretest probability, followed by R74 Upper respiratory infection acute, which accounted for 17.1% of all diagnoses with a pretest probability of 29.0%. Out of all new health issues, these 2 items comprised more than 70% (pretest probability, 73.4%) of the cases.

**Table 3:**
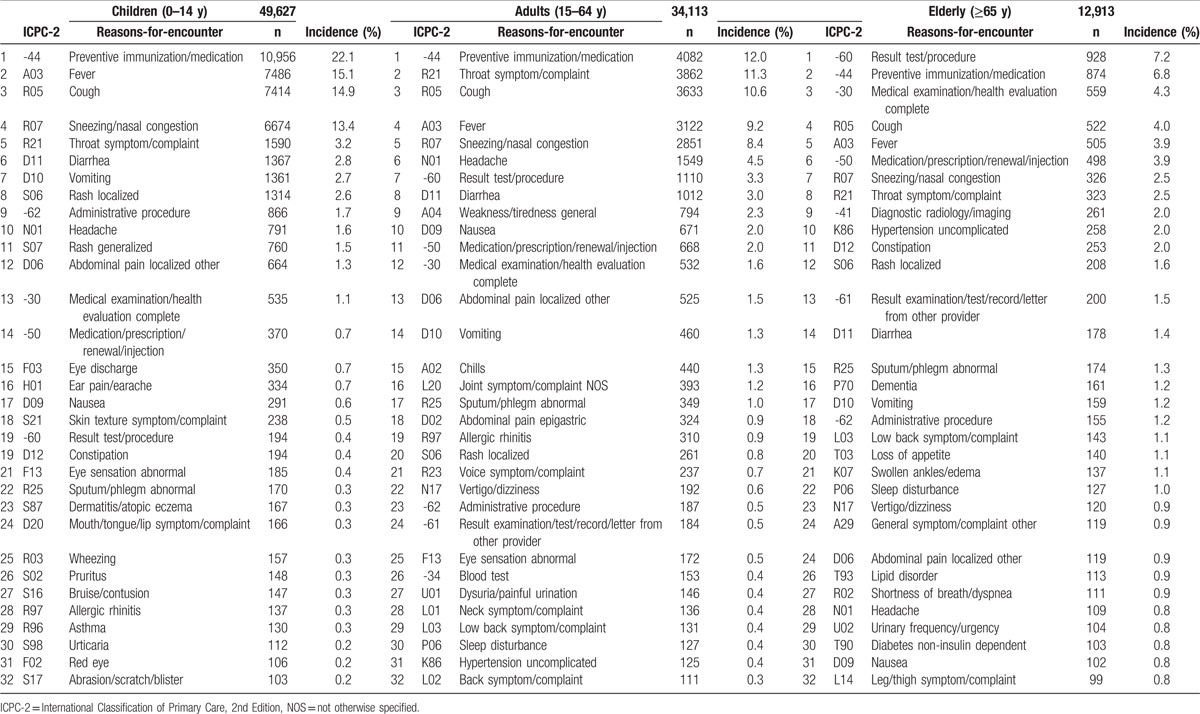
New reasons-for-encounter.

**Table 4:**
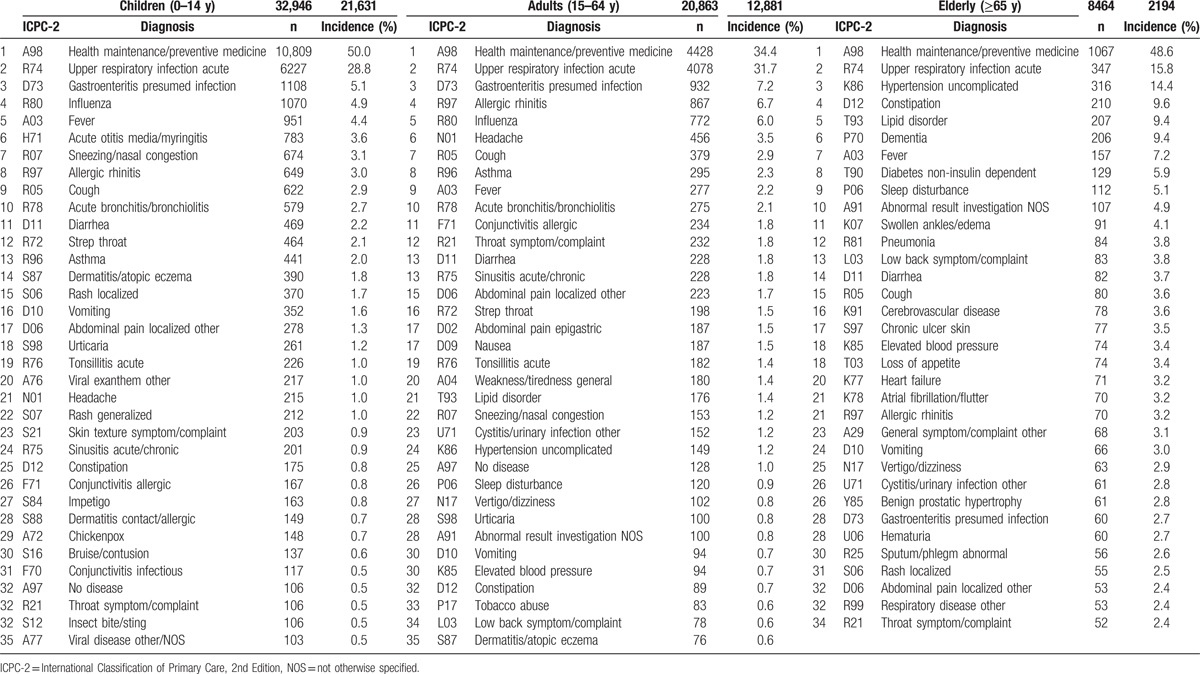
Final diagnosis.

### 3.3 Main results: (2) pretest probability with fever

The final diagnoses in the cases where A03 Fever was coded as a new reason-for-encounter are displayed in Table 5. In children and adults, R80 Influenza, D73 Gastroenteritis presumed infection, and R72 Strep throat, which are infection-related codes, comprised an overwhelming majority of the cases. In the elderly, R81 Pneumonia, P70 Dementia, D12 Constipation, and P06 Sleep disturbance were among the common codes that were not as common among children and adults.

**Table 5:**
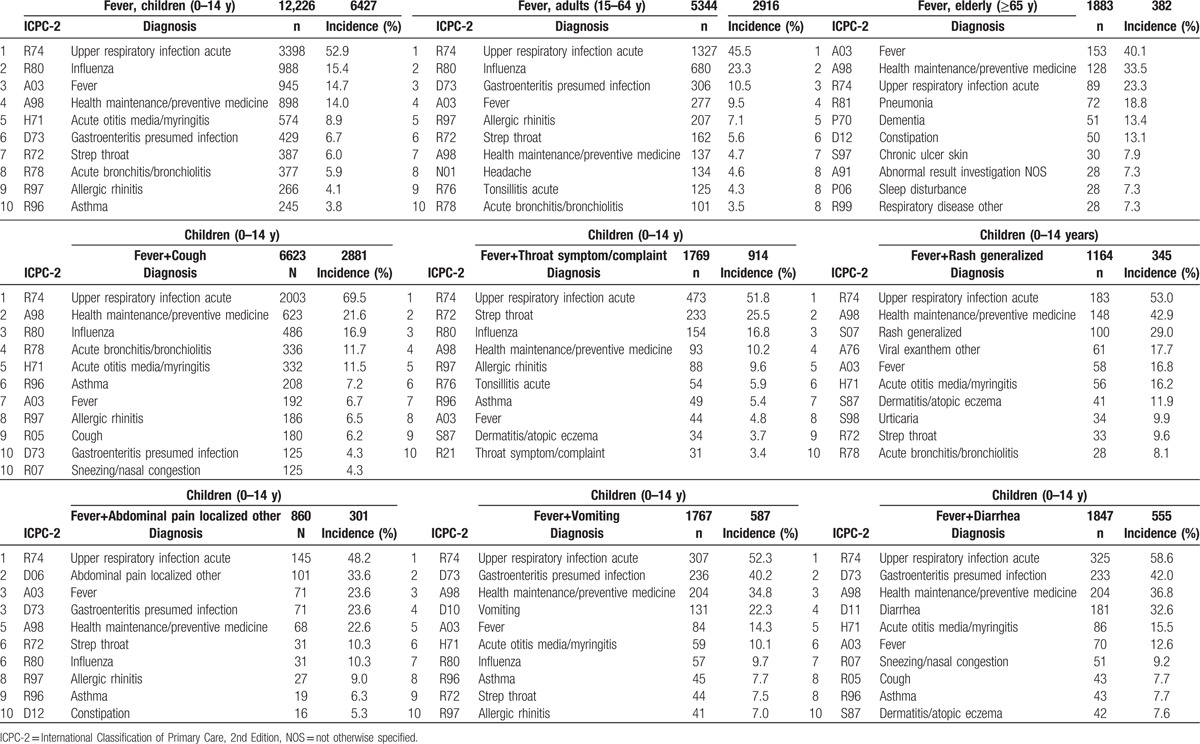
Final diagnosis of patients with fever.

Furthermore, regarding the final diagnosis, the conditional pretest probability coded A03 Fever (fevers of undetermined causation), was low in both children and adults at 14.7% and 9.5%, respectively, but was high in the elderly at 40.1%.

### 3.4 Other analyses

When considering the conditional pretest probability in children with fevers combined with other reason-for-encounter, we were able to determine how much the pretest probability changed based on the combined symptoms. For instance, compared with A03 Fever (6.0%) alone, R72 Strep throat combined with R21 Throat symptom/complaint (25.5%, LR+ 5.36) raised the conditional pretest probability. Moreover, compared with A03 Fever (15.4%) alone, when fever was accompanied by R80 Influenza combined with upper respiratory conditions such as R05 Cough (16.9%, LR+ 1.12) or R21 Throat symptom/complaint (16.8%, LR+ 1.11), the conditional pretest probability did not change; however, when A03 Fever was accompanied by codes related to the digestive system, such as D06 Abdominal pain localized other (10.3%, LR+ 0.63), D10 Vomiting (9.7%, LR+ 0.59), or D11 Diarrhea (7.0%, LR+ 0.41), the conditional pretest probability was decreased.

## 4 Discussion

### 4.1 Key results

This study is the first of its kind to report utilizing past accumulated diagnostic data in a clinic to calculate pretest probabilities in real time to assist in diagnosing patients. When we examined the new reasons-for-encounter, immunization, physical examination, and acute upper respiratory infections comprised the majority of reasons for seeing patients in all age groups. In the final diagnoses where the fever was a top new reason-for-encounter, influenza and other infectious diseases were the most common in children and adults. However, for the elderly, pneumonia, dementia, constipation, and sleep disturbances were found to accompany the fever; moreover, unlike other age groups, in 40% of cases, the fevers dissipated without ever determining the cause.

### 4.2 Interpretation and generalizability

There are prior studies on outpatients’ reasons-for-encounter,^[4–12]^ and we have found the same tendencies for the most frequent reasons-for-encounter or diagnoses. However, in these prior studies, the frequency of the reasons-for-encounter and the frequency of the final diagnosis were expressed independently and were not stratified based on age. The reason for this is that these prior studies were conducted to examine the range of health issues with which family practitioners deal in their daily practices.

Of these prior studies, only the study by Waza et al^[4]^ reported a comparison of the frequencies of the main reasons-for-encounter in a hospital's outpatient facility over a year as well as comparison by age. In that study, in 142 cases, including those in children aged 0 to 14 years, with A03 Fever as the reason-for-encounter, the diagnosis (ICD-10) in 58.5% of the cases was acute upper respiratory infection, acute gastroenteritis in 12.0%, and acute tonsillitis in 7.7%; influenza was not in the 10 most common diagnoses. In 16 cases where the patients were more than 65 years old, 31.3% were diagnosed as acute upper respiratory infection, while 18.8% had arthritis and 18.8% had fever of undetermined cause. The current results are similar to these prior results in that acute upper respiratory infection was more common in children and adults and having a fever of undetermined cause was common in the elderly. It is possible that in many cases of elderly in-home care patients developing fevers, management (experimental treatment) is prioritized without a definite diagnosis. However, there is room for further consideration regarding the phenomenon that the fever of such patients is often alleviated naturally. Regardless, the environment for outpatient care has changed since the prior study, and it is no longer as simple to compare cases such as influenza, as it has been underdiagnosed in Japan.

Although we only partially considered the final diagnosis (conditional pretest probability) of patients with fever, it became evident that the pretest probability fluctuates greatly based on age and reason-for-encounter. For example, children with gastrointestinal symptoms have been shown to have lower conditional pretest probability for influenza. This finding is consistent with that of a prior study^[15]^ suggesting that the probability of an influenza diagnosis decreases (odds ratio: 0.84) when gastrointestinal symptoms are observed. By using the accumulated medical data to correct the subjective diagnostic judgments of the physicians, we can expect to improve each individual diagnostic process.

Furthermore, to provide a more precise diagnosis and decisions regarding treatment plans, it is first necessary to further study the various factors influencing the diagnosis. CDSS can provide the foundational medical data for such a study.

For example, in the case of infectious diseases and other epidemic diseases, CDSS would likely be useful for epidemic forecasting and activities that could raise awareness. Moreover, in cases where there are vast differences among various medical care settings or among physicians, such as in the field of family medicine, comparing diagnostic characteristics could improve the quality of care as well as be helpful for further clinical studies. To assist with further clinical studies, we will continue to collect further data and conduct further analyses.

### 4.3 Limitations

This study has some structural limitations. First, all diagnoses depended on clinical diagnosis, so it was not possible to determine the accuracy of the diagnosis. When the patients revisited the clinic, we learned the outcomes after the fact; however, in the absence of such revisits, we would have been unable to obtain accurate information regarding the outcomes and may not have been able to accurately reflect the diagnoses of other hospitals and medical facilities that were also visited. Therefore, though the diagnostic accuracy increases with diseases with a higher rate of occurrence, in the case of rare diseases, diagnostic accuracy decreases due to lack of data. Accordingly, it is a challenge to improve the system to more accurately determine outcomes in clinical settings; nonetheless, it is important that, based on the characteristics of the system, it is not suitable for assisting in diagnosing rare diseases.

Furthermore, bias cannot be excluded during coding. Fever tends to be identified by caregivers and coded by the attending physician, thus creating detection bias. Biases such as gastroenteritis-presumed infection with respect to cases of diarrhea tend to be coded based on specific symptoms. Thus, it is possible that such biases may influence the data. The diagnostic tendencies of the attending physician can become a major source of bias because of the relatively short time allotted for coding within the total care time, and we would like to consider this type of bias in future studies.

Additionally, there is an instability issue with ICPC-2. For example, there are diseases, such as Kawasaki disease, that do not have corresponding reason-for-encounter or diagnostic codes (or the inability to enter detailed names of the diagnosis into the codes). Moreover, as in the case of P17 Tobacco abuse, some codes may or may not be coded depending on the attending physicians. Technological improvements of the interface to overcome and minimize such instabilities in the codes are challenges that lay ahead.

## 5 Conclusion

We reported the details of diagnoses, which utilized CDSS for the diagnostic process over a 4-year period for all 11,688 individuals who used the services of an urban clinic in Japan. This study illustrates it is possible to use CDSS to obtain epidemiological data such as the reasons-for-encounter and final diagnoses, as well as enabling calculation of the pretest probability based on age or reason-for-encounter. CDSS could be a useful tool in understanding and improving the reality and quality of the diagnoses.

## Acknowledgments

The authors would like to acknowledge the technical assistance of Yasuhito Kushida (system engineer, Macros Japan Co., Ltd.) and Hideki Kawamoto (CEO, Macros Japan Co., Ltd.) from Aug 2010 to May 2015.
